# 2358 Expanding our educational reach: Development of a massive open online course (MOOC)

**DOI:** 10.1017/cts.2018.210

**Published:** 2018-11-21

**Authors:** Nicole L. O’Dell, Eric Fredericksen, Sarah Peyre

**Affiliations:** 1 University of Rochester Medical Center; 2 University of Rochester Warner School of Education

## Abstract

OBJECTIVES/SPECIFIC AIMS: Translational Science 101 aims to: (1) Orient the public to the field of clinical and translational science; (2) Provide a brief overview of each phase of translation (T0-T4); (3) Provide real-world examples of clinical and translational researchers and research projects that have directly impacted patients; (4) Provide learners with information on how they can become involved in clinical and translational science through many different avenues (study volunteer, student, faculty member, or study coordinator). METHODS/STUDY POPULATION: The primary audience for Translational Science 101 is the general public and media outlets who are interested in learning more about clinical and translational science and how this research is improving population health. The University of Rochester Clinical and Translational Science Institute created the course in order inform the public about the field of clinical and translational science, orient the public to the types of research that fall under the translational science umbrella, and demonstrate how translational research impacts populations. The Coursera Massive Open Online Course (MOOC) platform was selected to host the course in order promote the greatest level of exposure and also to expand the educational reach of the UR-CTSI to new external audiences. The course was constructed from scratch utilizing the Community of Inquiry (CoI) framework, an approach that is often utilized to guide the design and construction of asynchronous online coursework. CoI highlights the elements of social presence, cognitive presence and teaching presence as key factors impacting the educational experience learners have when enrolled in an online course. Discussion boards, embedded quizzes, and end of module quizzes were integrated in to the course design to promote learner engagement, collaborative learning, and interactions among learners. The “storytelling” instructional strategy is the backbone of the Introduction to Clinical Science modules, with various researchers from the University of Rochester Medical Center explaining their lines of research and how the research impacts patients and communities. Educational research has shown that there are many benefits to including storytelling in instruction (Green, 2004; Geanellos, 1996), including: (1) Stories create interest: The narrative structure increases learner interest and engagement as they are drawn in to a good story. (2) Stories create a more personal link between the learner and the content: Storytelling allows exploration of shared lived experiences without the demands of practice and allows students to make connections between the shared experiences and their own previous experiences and knowledge. (3) Stories provide a structure for remembering course materials: The inclusion of stories facilitates remembering because it is easier to remember a story rather than a list of disparate facts, and stories evoke vivid mental images which are an excellent cue for recall. (4) Stories are a familiar and accessible form of sharing information: Storytelling aids in overall learner understanding as it is a nonthreatening way of sharing information. Storytelling can also enhance course discussions as students feel more at ease discussing a story than discussing abstract or new concepts that they are still in the process of mastering. RESULTS/ANTICIPATED RESULTS: Introduction to Translational Science was launched on October 16, 2017, and is automatically scheduled to begin a new session every 3 weeks. To date the course has reported the following analytics: (1) 2308 learners have visited the course page, (a)476 learners have enrolled in the course; (b) 244 learners are currently active in the course; (c) 11 learners have completed all of the requirements of the course. (2)Learners by Continent, (a) North America 31%; (b) Asia 30%; (c) Europe 23%; (d) Africa 9%;(e) South America 5%; (f) Oceania 2%. (2) Learners by Country: Learners have come from 84 different countries from around the world. The 15 highest enrollment numbers are: (a) USA 25%, (b) India 11%, (c) Egypt 3.7%, (d) United Kingdom 3.4%, (e) Mexico 3.2%, (f) Brazil 2.8%, (g) China 2.8%, (h) Saudi Arabia 2.2%, (i) Spain 2.2%, (j) Germany 1.7%, (k) Russian Federation 1.7%, (l) Malaysia 1.5%, (m) Turkey 1.5%, (n) Italy 1.5%, and (o) Canada 1.5%. (3) Gender: 48% women and 50% men. (4) Age: (a) 13–17: 0.72%, (b) 18–24: 19.6%, (c) 25–34: 44%, (d) 35–44: 14.4%, (e) 45–54: 8.6%, (f) 55–64: 7.2%, (g) 65+: 3.6%. (5)Highest Education Level o Doctorate Degree: 17%; (a) Professional School Degree: 14%; (b) Master’s Degree: 31%; (c) Bachelor’s Degree: 27%; (d) Associate’s Degree: 2.3%; (e) Some College But No Degree: 4.5%; (f) High School Diploma: 3.8%; (g) Some High School: 0.75%. DISCUSSION/SIGNIFICANCE OF IMPACT: The Massive Open Online Course (MOOC) platform offers new, exciting opportunities for CTSA institutions to create courses and trainings that are accessible by learners all over the world. This greatly expands the educational reach that the CTSA education programs can have, moving beyond hub-focused or consortium-focused education to a much broader audience. The expansion of educational reach can promote increased visibility of the CTSA program, encourage collaborations amongst researchers at different institutions, and also inform the public about clinical and translational science, potentially fostering advancement opportunities.

